# Delayed circadian phase is linked to glutamatergic functions in young people with affective disorders: a proton magnetic resonance spectroscopy study

**DOI:** 10.1186/s12888-014-0345-1

**Published:** 2014-12-11

**Authors:** Sharon L Naismith, Jim Lagopoulos, Daniel F Hermens, Django White, Shantel L Duffy, Rebecca Robillard, Elizabeth M Scott, Ian B Hickie

**Affiliations:** Clinical Research Unit, Brain & Mind Research Institute, The University of Sydney, Camperdown, NSW 2050 Australia

**Keywords:** Depression, Youth, Circadian, Sleep, Bipolar, Affective, Glutamate, Glx, Spectroscopy, Actigraphy

## Abstract

**Background:**

While the association between affective disorders and sleep and circadian disturbance is well established, little is known about the neurobiology underpinning these relationships. In this study, we sought to determine the relationship between a marker of circadian rhythm and neuronal integrity (N-Acetyl Aspartate, NAA), oxidative stress (glutathione, GSH) and neuronal-glial dysfunction (Glutamate + Glutamine, Glx).

**Methods:**

Fifty-three young adults (age range 15–33 years, mean = 21.8, sd = 4.3) with emerging affective disorders were recruited from a specialized tertiary referral service. Participants underwent clinical assessment and actigraphy monitoring, from which sleep midpoint was calculated as a marker of circadian rhythm. Proton magnetic resonance spectroscopy was performed in the anterior cingulate cortex (ACC). The metabolites NAA, GSH and Glx were obtained, and expressed as a ratio to Creatine.

**Results:**

Neither NAA or GSH were associated with sleep midpoint. However, higher levels of ACC Glx were associated with later sleep midpoints (rho = 0.35, *p* = 0.013). This relationship appeared to be independent of age and depression severity.

**Conclusions:**

This study is the first to demonstrate that delayed circadian phase is related to altered glutamatergic processes. It is aligned with animal research linking circadian rhythms with glutamatergic neurotransmission as well as clinical studies showing changes in glutamate with sleep interventions. Further studies may seek to examine the role of glutamate modulators for circadian misalignment.

## Background

It is well established that affective disorders are associated with sleep-wake and circadian disturbance [1-4]. Indeed, circadian dysfunction is postulated to play a role in the pathogenesis of mood disturbance onset, maintenance and recurrence [5-12]. In younger people with affective disorders, data on sleep-wake and circadian change is only beginning to emerge but suggests clear circadian misalignment early in the course of disease. Specifically, delayed sleep phase is prominent, with over 40% of young patients showing this profile [13]. In addition, advancing illness is associated with lower evening melatonin secretion, as well as short phase angles between melatonin onset and habitual sleep onset [14]. Both the unipolar and bipolar subtypes show delayed sleep and melatonin patterns, though some data indicates more pronounced phase shifts in those with bipolar disorder [4,13].

Despite the recent advances in treatments specifically focusing on the sleep-wake system [15-18], our understanding of the relationship between circadian change and underlying neurobiological processes is embryonic. Indeed, even in healthy adults or adolescents there is a dearth of research specifically examining the inter-relationships between such factors. Understanding these relationships early in the course of affective disorders is relevant not only to further our scientific understanding of sleep-wake and circadian change but also to inform targeted intervention approaches [15]. Using proton magnetic resonance spectroscopy (^1^H-MRS) we have previously reported that within the anterior cingulate cortex (ACC, a key region implicated in affective disorders), the neurometabolites N-Acetyl Aspartate (NAA; a neuronal integrity marker), glutamate (an excitatory neurotransmitter) and glutathione (GSH; a marker of oxidative stress) are likely to differ markedly across patient clusters despite their similar levels of current symptoms and functional impairments [17]. These findings are aligned with studies showing altered levels of these neurometabolites in affective disorders [19-22] and additionally suggest that consideration of these markers is warranted to better understand specific phenotypes, including those with prominent sleep-wake change. In this study, we sought to extend our work by examining sleep-wake and circadian functioning in relation to *in-vivo* measures of the brain neurometabolites NAA, GSH and glutamate + glutamine (Glx). We hypothesised that delayed sleep would be associated with decreased concentrations of NAA (indicative of neuronal compromise), GSH (indicative of oxidative stress), and increased Glx (indicative of excitatory neurotransmission).

## Methods

### Participants

Fifty-three young adults (age range 15–33 years, mean = 21.8, sd = 4.3) were recruited from specialised early intervention services for youth mental health (Youth Mental Health Clinic and *headspace*), Sydney, Australia [23]. Participants were a subset of a broader sample [17] participating in detailed neurobiological assessments. They were specifically selected for this study if their psychiatrist considered them to have an emerging affective (unipolar or bipolar) disorder [23] and they completed actigraphy monitoring and a magnetic resonance imaging scan within a one-month period. In addition to our usual exclusion criteria [17], participants undertaking shift work or having transmeridian travel within 60 days previous to data collection were excluded. Participants with an alcohol and/or substance dependence disorder were also excluded. All participants or their parents gave written informed consent prior to participation in the study. The study protocol was approved by the University of Sydney Human Research Ethics Committee.

### Procedure

#### Clinical assessment

As described elsewhere [23], patients were receiving care for their mental health disorder by a mental health professional and a clinical psychiatrist (namely ES, IH) confirmed affective disorder diagnoses. In addition, all participants were assessed by a trained research psychologist who recorded depression and mania symptom severity using the 17-item Hamilton Depression Rating Scale (HAM-D) [24]. Manic symptoms were rated for a subset of 39 participants using the Young Mania Rating Scale (YMRS) [25]. Twenty participants were taking antidepressant medications at the time of assessment which included nine taking selective serotonin reuptake inhibitors, seven taking serotonin noradrenaline reuptake inhibitors, one taking a monoamine oxidase inhibitor, one taking a noradrenergic and specific serotonergic antidepressant, one taking a melatonergic antidepressant and one taking an atypical antidepressant. In addition, 11 participants were taking mood stabilising medications and 12 were taking antipsychotic medications. Thirty-two patients were classified as emerging unipolar and 21 as emerging bipolar disorder.

#### Sleep-wake ambulatory assessment

Participants completed sleep diary and actigraphy monitoring (Actiwatch-64/L/2, Philips Respironics, USA) for 14 days (mean duration = 12.2 days, sd = 3.3 days; median duration = 13.0 days) within one month of undergoing ^1^H-MRS. Actiware 5.0 software (Philips Respironics) was used to score actigraphy using a wake threshold value of medium sensitivity (40.0 activity counts/epoch). Based on sleep diaries, corrections by trained technicians were applied where necessary. Rest intervals were subsequently submitted to dual integration to define periods spent awake and resting. For the purpose of this study, actigraphic measures will be defined as ‘sleep'. The following actigraphic sleep variables were obtained for descriptive purposes: ‘sleep onset’ and ‘sleep offset’ (mean timing of the onset/offset of the rest intervals, respectively), time in bed (TiB: total duration of the rest intervals), total sleep time (TST: amount of time scored as “sleep” within the rest episode), ‘wake after sleep onset’ (WASO: amount of time scored as “wake” within the rest episode), and sleep efficiency (‘total sleep time’/‘time in bed’)*100). The primary outcome of this study was the ‘sleep midpoint’, defined as “TST/2 + sleep onset”. Sleep midpoint has been found to be the most reliable indicator of melatonin onset in young healthy participants [26] with data showing moderate to high correlations with Dim Light Melatonin Onset [27]. Thus, sleep midpoint is a useful proxy marker for circadian phase when actigraphy and sleep diaries are utilised.

#### Proton magnetic resonance spectroscopy

**Figure 1:**
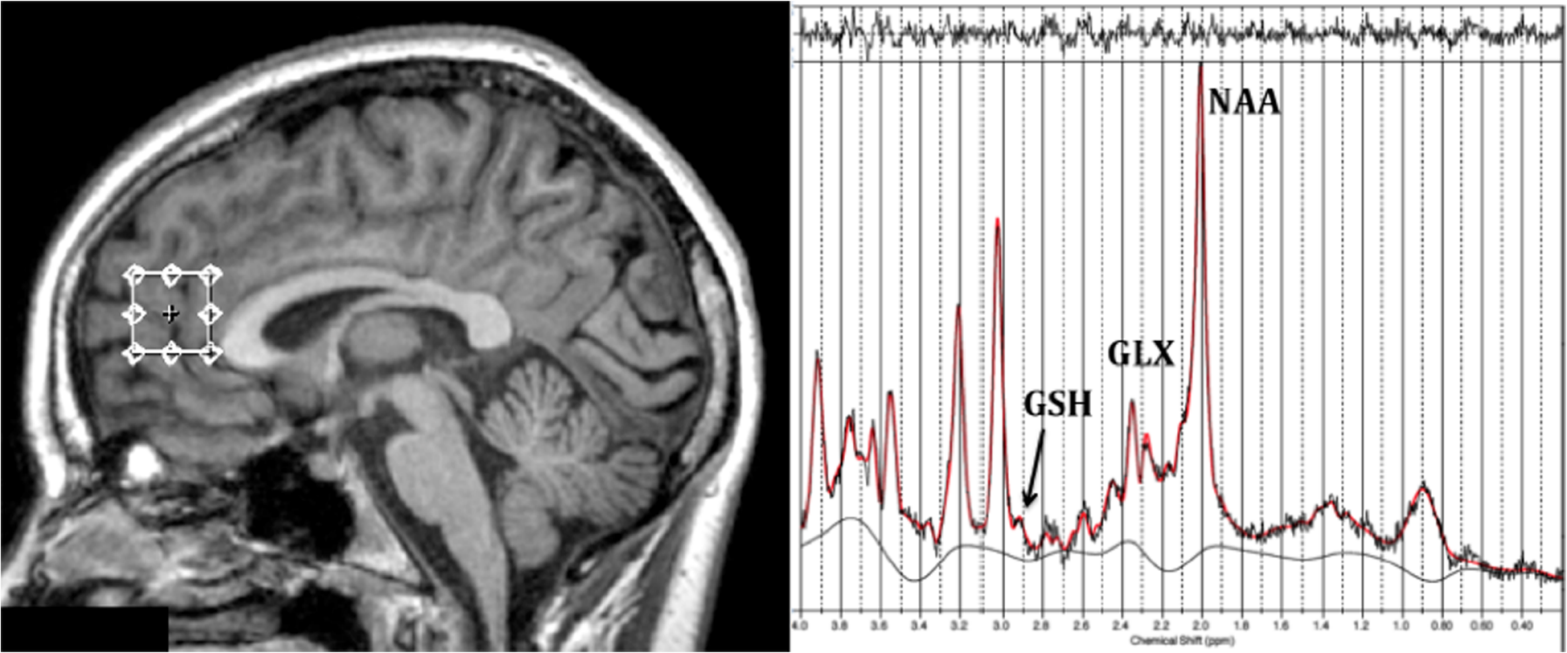
**Example anterior cingulate cortex voxel placement and resulting proton magnetic resonance spectroscopy spectra for one participant.** The spectra also shows Glutathione, Glutamate + Glutamine and N-Acetyl Aspartate peaks. Abbreviations are as follows: GSH = Glutathione; NAA = N-Acetyl Aspartate; Glx = Glutamate + Glutamine.

As described elsewhere [17,28], imaging took place on a 3-Tesla GE Discovery MR750 scanner (GE Medical Systems, Milwaukee, WI) using an 8-channel phased array head coil. The following images were acquired in order: (a) Three-dimensional sagittal whole-brain scout for orientation and positioning of subsequent scans; (b) T1-weighted magnetization prepared rapid gradient-echo sequence producing 196 sagittal slices to aid in the anatomical localisation of sampled voxels; and, (c) Single voxel ^1^H-MRS using Point RESolved Spectroscopy (PRESS) acquisition, with two chemical shift-selective imaging pulses for water suppression. Spectra were shimmed to achieve full-width half maximum of less than 13Hz. For each participant, a spectra was acquired from a voxel measuring 20×20×20 mm placed midline in the ACC (Figure 1; TE = 35 ms, TR = 2000 ms and 128 averages).

Following MRS acquisition, data was transferred offline for post processing using the *LCModel* software package [29] and metabolite concentrations were determined as a relative ratio to Creatine (Cr). All spectra were quantified using a PRESS TE = 35 basis set of 15 metabolites. Neurometabolites of interest were NAA, GSH and Glx. The spectra were visually inspected separately by two different raters (SD, JL) to ensure consistent spectra and poorly fitted metabolite peaks (as reflected by large Cramer–Rao lower bounds greater than 20%) were excluded from further analysis.

#### Statistical analysis

Data were analysed using the Statistical Package for the Social Sciences (version 20 for Mac). Student’s t-tests were used to compare group (unipolar *vs.* bipolar) data. Pearson’s or Spearman’s correlations were used for continuous data as appropriate. Analyses were two tailed and used an alpha level of 0.05.

## Results

Table 1 shows descriptive demographic, clinical, actigraphy and neurometabolite data for the sample. On average, the sample was 22 years of age with mild depressive symptoms. The mean sleep midpoint occurred at 4:14 am but ranged from 1:54 am to 7:05 am (median = 4:08 am). There were no differences between unipolar and bipolar participants in terms of age (t = −0.29, *p* = 0.771), gender, (*x*^2^ = 1.52, *p* = 0.217) depressive symptoms (HAM-D, t = 0.86, *p* = 0.395), neurometabolite concentrations (Glx, t = 0.14, *p* = 0.173; GSH, t = 1.15, *p* = 0.255; NAA, t = −0.26, *p* = 0.797) or sleep midpoint (t = 1.1, *p* = 0.915). As expected, YMRS total score was significantly higher in bipolar participants (t = −2.2, *p* = 0.034). Importantly, there were also no significant differences between patients taking mood stabilising or antidepressant medication compared to those not taking medication at the time of assessment in terms of age, gender, depressive symptoms, mania symptoms, neurometabolite concentrations (Glx, GSH, NAA) or sleep midpoint (all *p* > 0.05).

**Table 1:** **Demographic, clinical, actigraphic and MRS characteristics of participants (n = 53)**

**Characteristic**	**Mean ± SD**
Gender, % female	71.7 (38/53)^#^
Age, years	21.8 ± 4.3
Hamilton depression rating scale	13.3 ± 7.0
Young Mania Rating Scale^a^	3.6 ± 5.3
*Actigraphy*	
Sleep onset, time	23:50 ± 1:12
Sleep offset, time	08:37 ± 1:18
Total sleep time, hours	7:28 ± 0:30
Wake after sleep onset, mins	78.5 ± 25.6
Sleep efficiency, %	85.2 ± 4.2
Sleep midpoint, time	04:14 ± 1:09
*Anterior Cingulate neurometabolites*	
N-Acetyl Aspartate (NAA)/Creatine	1.29 ± 0.13
Glutathione (GSH)/Creatine	0.45 ± 0.12
Glutamate/Glutamine (Glx)/Creatine	2.07 ± 0.32

^#^Data represents percentage and number.

^a^Available in a subset of 39 participants.

**Figure 2:**
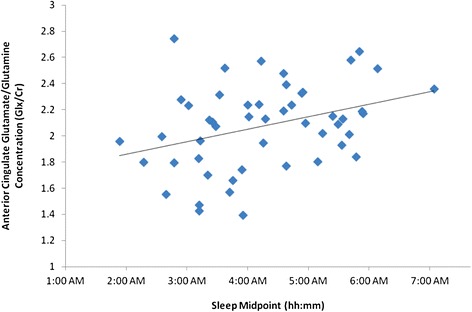
**Scatterplot showing the correlation between anterior cingulate Glx/Cr ratio and sleep midpoint in young persons with affective disorders.** Abbreviations are as follows: Glx = Glutamate + Glutamine; Cr = Creatine.

The mean time of MRS collection was 3:42 pm (sd = 1.8 hrs). Correlations between ^1^H-MRS neurometabolites and sleep midpoint showed that neither NAA or GSH were associated with sleep midpoint (r = 0.032, *p* = 0.825; r = 0.211, *p* = 0.151 respectively). However, as shown in Figure 2, higher levels of ACC Glx were associated with later sleep midpoints (rho = 0.35, *p* = 0.013). In order to examine whether this association was confounded by changes in the neurobiological and/or circadian system due to the developmental process or to depressive state phenomena, we conducted further analyses controlling for age and HAM-D score. The resultant significant partial correlations showed this relationship was robust (age, r = 0.36, *p* = 0.012; HAM-D, r = 0.37, *p* = 0.009). However, when examining the relationship between ACC Glx and sleep midpoint controlling for symptoms of mania (i.e. YMRS scores, n = 39), the resulting partial correlation was not significant (partial r = 0.06, *p* = 0.730). This is despite the fact that YMRS scores alone were not associated with ACC Glx (r = −0.29, *p* = 0.862). Thus, these latter findings may be reflective of the smaller sample with YMRS scores or alternatively may suggest that manic symptoms mediate the relationship between elevated ACC Glx and later sleep midpoint.

For descriptive purposes, we secondarily examined the association between Glx and key sleep parameters. While the association with TST, TiB and sleep onset were not significant (r = −0.03, *p* = 0.812; r = 0.10, *p* = 0.457; and r = 0.24, *p* = 0.083 respectively), Glx was significantly associated with sleep offset (r = 0.30, *p* = 0.030), WASO (r = 0.325, *p* = 0.017), and sleep efficiency (r = −0.31, *p* = 0.023).

## Discussion

This study is the first to demonstrate an association between *in vivo* neurobiological measures of brain neurometabolites and delayed sleep phase in young persons with affective disorders. Specifically, higher levels of ACC Glx, a surrogate marker of the glutamatergic system, were associated with a later sleep midpoint, suggestive of delayed circadian phase. Markers of neuronal integrity and oxidative stress were not associated with circadian phase in this sample.

In this study of youth with emerging affective disorders, we did not find any group differences in neurometabolites or sleep variables between those participants considered by the psychiatrist to have either a unipolar or bipolar illness. While the reasons for this are unclear, it is possible that they reflect the fact that the symptom severity of this sample was rather mild and encompassed those with emerging rather than full-threshold illnesses. That is, even those that were considered to have a ‘unipolar’ illness may eventually progress to bipolar disorder with longitudinal follow-up. Certainly, our data in the sub-sample of 39 participants with detailed measurement of symptoms of mania suggests that manic symptoms may contribute to the association between delayed circadian phase and Glx. Thus, it is possible that those on a trajectory to bipolar disorder are accounting for the major findings of this study, and future studies focusing on the specificity of these findings within the affective disorder sub-types is warranted.

These findings focussing on neurometabolites within the ACC are aligned with those implicating the ACC in the neurobiology of depression [30]. The ACC is densely interconnected with the hippocampus, dorsolateral prefrontal cortex, amygdala, orbitofrontal cortex and hypothalamus and therefore these findings may also reflect the involvement of broader neuronal circuitry. Prior work has shown this structure to be linked to cognitive impairment and other phenotypic features in affective disorders [17,31]. However, this study is the first to show that neurometabolite concentrations within the ACC are also relevant to sleep-wake functions. While the primary objective of this study was to examine circadian rhythm, secondary analyses also showed that higher levels of ACC Glx were related to nocturnal wakefulness and poorer sleep efficiency. Unfortunately, even in healthy samples, little is known about the inter-relationships between ACC, mood and sleep-wake functions. However, recent data from functional imaging studies show that there are diurnal changes in regional cerebral blood flow within the ACC [32], with the ACC showing a strong circadian bias in the morning. Additionally, this region is consistently implicated in insomnia (see review by Speigelhalder [33]). Further studies are now needed to further explicate how changes within the ACC circuitry may relate to circadian misalignment and nocturnal wakefulness in affective disorders.

While there is currently a dearth of studies that have examined the association between circadian and spectroscopic markers in depressive disorders, the findings of this study suggest that the glutamatergic system may be implicated in the pathogenesis of this phenotypic feature. There are certainly documented dense glutamatergic projections from the suprachiasmatic nucleus (SCN), via the ventral subparaventricular zone and dorsomedial hypothalamic nucleus, to the sleep-wake centers of the lateral hypothalamus, thereby regulating sleep-wake timing [34]. Glutamate is also a neurotransmitter of the optic nerve and retinohypothalamic tract that the SCN is sensitive to and this neurotransmitter does appear to phase shift circadian rhythms [35,36].

With regard to the sleep system more generally, two studies have shown changes in Glx in association with sleep interventions. Specifically, Benedetti et al. [37] reported that levels of ACC Glx decrease in association with sleep deprivation, and light therapy was associated with improvements in depressive symptoms. Accordingly, Murck et al. [38] found that for males with depression, sleep deprivation is associated with an increase in Glx. The specific role that Glx substituents assume in sleep-wake cycles is currently unknown. However, it is clear that glutamate signalling is critically involved in information transmission, plasticity and neurotoxicity. Animal studies show that it increases during both waking and rapid eye movement sleep states and when the sleep period is preceded by wake states [39,40]. Additionally, the rate of glutamate decrease is greatest with sleepiness and when slow wave activity is high [39]. Thus, it is feasible that sleep-wake history mediates glutamatergic signalling in affective disorders, though such causative relationships are yet to be demonstrated in humans.

While this study represents the first to examine the association between delayed circadian rhythms and underlying neurobiology in youth depression, several methodological factors should be considered when interpreting our findings. Firstly, the lack of a control group is a limitation of this study. This would have been particularly helpful for interpretation of these findings since it is currently unclear how sleep midpoint relates to ACC Glx in healthy adolescent or even adult samples. Secondly, adolescence itself is known to be associated with circadian delay [41]. Thus, from these results, we cannot ascertain whether our findings are reflective of adolescence generally or whether they are specific to affective disorders. Third, although the sample size of 53 is not particularly small for these types of detailed neurobiological studies, this sample is rather heterogeneous since ultimate illness trajectory is unknown in these early clinical stages [42]. Also, a larger sample would have been optimal in terms of conducting multivariable analyses whereby the influence of potential confounds could be determined concurrently. In terms of other sample characteristics, it is also important to note that details regarding female study participants’ menstrual cycle phase were not collected. Increasing evidence suggests that menstrual phase affects sleep in females [36]. However we were unable to account for the possible influence of this factor. Another consideration that could have certainly influenced circadian rhythm is psychotropic medication [43] and larger datasets would be required to analyse the contribution of antidepressant, mood stabilising or anti-psychotic medication.

Regarding the spectroscopy component of the study, it is important to note that since we examined Glx as a surrogate marker of glutamate, the current findings may in part be reflective of glutamine. Further research examining the roles of Glu and glutamine are now required to further delineate their role in sleep-wake function in both healthy and psychiatric samples. Furthermore, this study used a PRESS sequence to examine GSH concentration. Although we have previously validated the GSH signal measured using this sequence, replication of null findings using MEGA-PRESS may be warranted. In this study, MRS measures were derived across various times of the day, and if *in-vivo* measures of brain neurometabolites also vary according to circadian rhythm, this is a potential source of noise that could be eradicated if scanning were to occur at a fixed time period. Finally, due to the inability to conduct spectroscopy within the SCN or its neighbouring hypothalamic sleep-wake centres, we were of course unable to measure Glx within these regions. Thus, we cannot be confident that ACC Glx sufficiently captures glutamatergic concentration within regions critical for the circadian regulation of sleep.

In terms of sleep and circadian assessment, this study used actigraphy to derive sleep midpoint, but polysomnography studies may yield a more accurate determination of this measure and future investigations should incorporate melatonin and other endogenous circadian markers.

## Conclusions

Overall, this study is the first to demonstrate a link between markers of the glutamatergic system in affective disorders and delayed sleep-wake cycle. It enhances our prior work examining delayed sleep and melatonin in affective disorders [4,13,14] by specifically linking this key phenotypic feature to a neurobiological marker implicated in affective disorders, and provides an impetus for further exploratory work in this area. Our results further suggest that this association might be particularly influenced by symptoms of mania. While not the focus of this study, we also found that ACC Glx was associated with poorer sleep, as evidenced by more nocturnal awakenings and poorer sleep efficiency.

Whilst these findings are preliminary and require replication in larger samples, they have potential significance for the clinical management of young people with emerging affective disorders. In particular, given the increasing recognition of the role of glutamate in affective disorders, it is possible that glutamatergic modulators might help to restore circadian misalignment, which in turn, could prevent affective symptom onset, severity and recurrence [15].

## Abbreviations

^1^H-MRS: Proton magnetic resonance spectroscopy
ACC: Anterior cingulate cortex
Cr: Creatine
Glx: Glutamate + Glutamine
GSH: Glutathione
HAM-D: Hamilton depression rating scale
NAA: N-Acetyl Aspartate
SCN: Suprachiasmatic nucleus
TiB: Time in Bed
TST: Total sleep time
WASO: Wake after sleep onset
